# Clinical outcomes at 6 months after MRgFUS thalamotomy in Parkinson’s disease: a large longitudinal cohort study

**DOI:** 10.1007/s00415-026-14021-7

**Published:** 2026-07-26

**Authors:** Francesca Pistoia, Federico Salfi, Francesco Arrigoni, Gennaro Saporito, Federico Bruno, Alessia Catalucci, Maria Letizia Pistoia, Simone Cesarano, Simona Sacco, Alessandra Splendiani, Patrizia Sucapane

**Affiliations:** 1 Department of Biotechnological and Applied Clinical Sciences, University of L’Aquila, L’Aquila, Italy; 2 Department of Neurology, San Salvatore Hospital, L’Aquila, Italy; 3 Department of Radiology, San Salvatore Hospital, L’Aquila, Italy

**Keywords:** Parkinson’s disease, Tremor, MR-guided focused ultrasound, Thalamotomy

## Abstract

**Background:**

Magnetic resonance–guided focused ultrasound (MRgFUS) is an expanding therapy for tremor in Parkinson’s disease (PD). This study aimed to characterize trajectories of tremor and cognitive functions following MRgFUS treatment over 24 hours, 1 month, and 6 months, and to explore potential predictors of outcomes.

**Methods:**

This single-center, prospective, observational cohort study included PD patients undergoing MRgFUS over a 7-year period. Tremor severity was assessed using the Fahn–Tolosa–Marin Tremor Rating Scale (FTM) and the Movement Disorder Society Unified Parkinson’s Disease Rating Scale Part III (MDS-UPDRS-III). Cognitive performance was evaluated using the Mini-Mental State Examination (MMSE) and the Montreal Cognitive Assessment (MoCA).

**Results:**

107 patients were included. Tremor severity showed a significant time effect (FTM and MDS-UPDRS-III: both p < 0.001), with a marked reduction at 24 hours followed by partial attenuation and stabilization over time, while remaining lower than baseline at 1 and 6 months (both p < 0.001). The 24-hour improvement was greater in patients with longer disease duration, who showed higher baseline severity (p < 0.001) and converged to cohort-level severity at post-treatment timepoints (all p ≥ 0.085). Cognitive status (MMSE and MoCA scores) remained stable throughout follow-up (all p ≥ 0.287). Levodopa equivalent daily dose (LEDD) significantly decreased from baseline to 6 months (F = 19.992, p < 0.001), suggesting a reduced need for dopaminergic treatment following tremor improvement.

**Conclusions:**

MRgFUS provided meaningful tremor improvement without negative effects on cognition. The significant LEDD reduction at 6 months supports a beneficial medication-sparing effect associated with tremor improvement.

## Introduction

Magnetic Resonance-guided Focused Ultrasound (MRgFUS) is a well-established yet rapidly evolving non-invasive therapeutic option for movement disorders, particularly essential tremor (ET) and Parkinson’s disease (PD) [1, 2]. Recent research highlights its growing popularity, driven by increasing precision and improved clinical outcomes, enabling patient-centered management and enhanced quality of life [3]. One of the main advantages of MRgFUS over other surgical techniques is its ability to create a highly targeted lesion, with adverse events that are rare, mild, and transient, along with measurable efficacy both in clinical tests and in the patient's daily life. Compared to other surgical techniques such as deep brain stimulation (DBS), MRgFUS represents a promising alternative, due to its non-invasive nature and lower procedural risks. However, MRgFUS is an ablative technique limited to tremor control, whereas DBS modulates a broader spectrum of motor symptoms.

Previous clinical studies support the efficacy of MRgFUS, with limited availability of outcome data [2, 4–8]. The strongest evidence comes from a randomized, double-blind, sham-controlled trial in patients with tremor-dominant PD, demonstrating the superiority of active treatment over the sham procedure, while maintaining a favorable safety profile [6]. These findings were further supported by a prospective open-label study [7] and a comprehensive systematic review [8], both confirming the effectiveness and favorable safety profile of MRgFUS in patients with tremor-dominant PD. Another critical issue in MRgFUS treatment, especially in PD compared with ET, is the risk of symptom relapse over time after treatment. Relapses are influenced by both modifiable and non-modifiable factors. Lesion location and volume are the main predictors of long-term outcomes, with deeper and precisely targeted lesions associated with fewer relapses and better quality of life [9, 10]. Cognitive performance following MRgFUS also warrants further investigation, particularly in the context of emerging bilateral treatment approaches, currently supported by limited evidence [4, 11, 12]. Therefore, identifying factors that influence MRgFUS efficacy and predict outcomes is essential to guide patient management across different therapeutic options and to consider bilateral treatments in carefully selected cases.

The aim of this study was to characterize tremor trajectories and to identify predictors of outcome in patients treated with MRgFUS over a 6-month follow-up period.

## Methods

### Study design and participants

This is a prospective, single-center, observational, real-word registry including all consecutive patients with PD undergoing MRgFUS for PD-related tremor between January 2018 and December 2024 at the Neurology and Stroke Unit, San Salvatore Hospital, L’Aquila, Italy.

PD diagnosis was performed by movement disorder specialists (FP, PS) using the Movement Disorder Society PD Criteria [13, 14]. According to the clinical protocol, patients with PD treated with MRgFUS were included in the study if the following inclusion criteria were satisfied: (i) patients undergoing MRgFUS for severe, medically refractory tremor, significantly impairing activities of daily living, ii) patients receiving a technically successful procedure (a reached temperature within the nidus ≥55°C, achieving an effective ablation [15]), and (iii) patients with complete pre-treatment clinical data (age, sex, disease characteristics, including disease duration) and available baseline Fahn–Tolosa–Marin Clinical Rating Scale for Tremor (FTM) scores [16]. Patients with additional neurological disorders or psychiatric symptoms, other central neurodegenerative diseases other than PD, secondary parkinsonism, presence of dementia or intellectual disability, alcohol/drug abuse or incomplete clinical data and procedural reports were excluded.

A structured case-report form was used to collect demographic and clinical data (including age, sex, disease characteristics and duration) as well as procedural variables (including sonication parameters and skull density ratio [SDR]).

This report follows the STROBE checklist for reporting observational studies [17].

### Clinical assessment

Patients were evaluated at baseline, 24 hours, 1 month, and 6 months through the FTM [16] and the Movement Disorder Society Unified Parkinson’s Disease Rating Scale Part III (MDS-UPDRS-III) [18]. Tremor was assessed in the medication-OFF state to better isolate the treatment effect, minimizing the confounding influence of drugs and allowing a more accurate evaluation of the benefit attributable to MRgFUS. A subsample also completed the Mini-Mental State Examination (MMSE) [19], which was introduced after study initiation. Finally, the Montreal Cognitive Assessment (MoCA) [20] was administered at baseline and at the 1- and 6-month follow-ups.

### Neuroradiological procedure and parameters

All procedures were performed as previously explained [21]. Briefly, the ablation target was the ventral intermediate nucleus (VIM) of the thalamus, contralateral to the side of predominant clinical symptoms. Indirect coordinates were determined on MRI images as follows: along the anteroposterior axis, the target was located halfway between one-third and one-fourth of the anterior commissure–posterior commissure (AC–PC) distance from the PC (axial plane); along the mediolateral axis, halfway between 14 mm from the AC–PC line and 11 mm from the lateral wall of the third ventricle (axial plane); and 2 mm above the AC–PC line along the craniocaudal axis. Once the target was identified, focused ultrasound energy was delivered until an ablative temperature was achieved. Ablation was performed only after target confirmation through clinical testing, indicated by transient tremor suppression during the delivery of sub-ablative energy.

### Outcomes

The primary outcome was a change in tremor severity evaluated through the FTM [16] and the MDS-UPDRS-III [18] at baseline, 24 hours, 1 month and 6 months.

Secondary outcomes were changes in cognitive scores as evaluated through the MMSE [19] and the MoCA [20].

Adverse events associated with MRgFUS were prospectively recorded according to our institutional clinical protocol throughout the procedure and during follow-up visits. Adverse events were classified into two categories according to their presumed underlying mechanism: (1) MRI/ultrasound-related adverse events, associated with the procedural environment (e.g., prolonged MRI positioning and ultrasound energy delivery), and (2) thalamotomy-related adverse events, associated with the creation of the thalamic lesion. MRI/ultrasound-related adverse events included dizziness, scalp burning, headache, and vasovagal reactions, whereas thalamotomy-related adverse events included paresthesias, contralateral weakness, gait instability, and dysgeusia. For each adverse event, the occurrence, timing, and clinical course were documented. The persistence or resolution of symptoms was assessed at each scheduled follow-up visit, allowing adverse events to be classified as transient or persistent.

### Statistical analysis

The cohort size was determined by the number of eligible consecutive patients undergoing MRgFUS during the study period, and no a priori sample size calculation was performed. Descriptive statistics were used to summarize baseline demographic and clinical characteristics. Continuous variables were reported as mean ± standard deviation (SD), while categorical variables were presented as frequencies.

To investigate longitudinal changes in the primary motor outcomes (the FTM and MDS-UPDRS-III) we employed linear mixed-effects models (LMMs). This approach was selected for its robustness in handling unbalanced data due to missingness and its ability to model both fixed effects and individual variability over time. In each model, *Time* (baseline, 24 hours, 1 month, and 6 months) was entered as a categorical fixed effect to assess both short- and long-term clinical trajectories. A random intercept for subject was included to account for the within-subject correlation of repeated measurements. All models were adjusted for key baseline covariates: *age*, *sex*, *disease duration*, *treated hemisphere*, and *SDR*. Additionally, potential moderation effects on motor outcome trajectories were explored by including interaction terms between *Time* and each covariate.

To further clarify the contribution of specific motor domains to changes in the MDS-UPDRS-III total score, additional LMMs were performed on MDS-UPDRS-III subdomain scores, including tremor, rigidity, bradykinesia, and axial symptoms. The same model structure used for the MDS-UPDRS-III total score was applied to each subdomain.

Furthermore, given the relevance of lesion-related and procedural factors for MRgFUS outcomes, additional exploratory LMMs were performed to examine whether procedural parameters and lesion volume moderated motor outcome trajectories. Total number of sonications, maximum energy, maximum power, maximum temperature, and lesion volume were entered separately in additional models, including both their main effect and interaction with Time, to avoid model overfitting and multicollinearity among procedural variables.

The same LMM framework was applied to secondary cognitive outcomes. For the MMSE, analyses were restricted to the subset of participants with available cognitive data at baseline. For the MoCA, models were fitted using the three available timepoints (baseline, 1 month, 6 months).

To complement mean longitudinal trajectories, we performed a complete-case responder-maintenance analysis. Clinically meaningful response was defined as a ≥30% reduction from baseline score. Among patients with complete data at baseline, 24 hours, 1 month, and 6 months who achieved clinically meaningful response at 24 hours, we evaluated the proportion maintaining this response at 1 and 6 months. Response rates at individual timepoints were also calculated among patients with available data at each assessment.

To address possible changes in dopaminergic treatment over follow-up, levodopa equivalent daily dose (LEDD) was calculated at baseline and at 6 months when medication data were available. LEDD values were derived using standard levodopa-equivalent conversion factors. Baseline and 6-month LEDD values were compared using a LMM with Time as fixed effect and a random intercept for subject.

All analyses were performed in R version 4.1.2 (R Foundation for Statistical Computing, Vienna, Austria), and LMMs were estimated using the *GAMLj* module (version 3.0.0). Models were fitted using restricted maximum likelihood, and p-values were computed based on the Satterthwaite approximation for degrees of freedom [22]. When a significant effect was found on categorical variables, Holm-adjusted pairwise post hoc comparisons were conducted using the *emmeans* R package. In the case of significant interactions with continuous variables, simple slope analyses and simple effect contrasts were conducted at three levels of predictor (estimated marginal mean – EMM, EMM ± 1 SD). All tests were two-tailed, with the significance threshold set a priori at p < 0.050.

## Results

### Sample composition

**Fig. 1.**
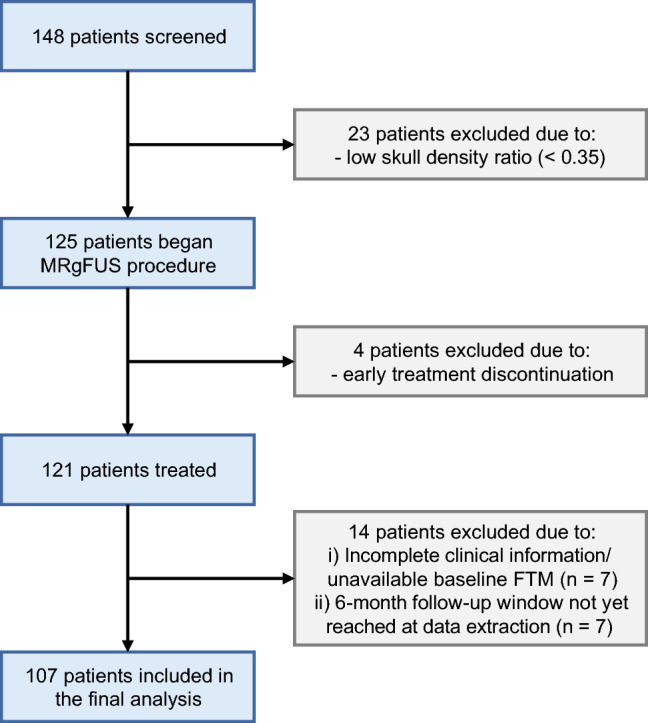
Patient selection flowchart

A total of 148 patients were screened during the study period; 23 were excluded because of an unfavorable SDR (< 0.35), resulting in 125 patients who were scheduled for MRgFUS and began the procedure; 18 were excluded because of (i) early treatment discontinuation (n = 4) due to claustrophobia or inability to maintain the required treatment position for the duration of the procedure, (ii) incomplete clinical information or unavailable FTM score (n = 7), or (iii) because the 6-month follow-up window had not yet been reached at the time of data extraction (n = 7), resulting in 107 patients included in the final analysis (Fig. 1). As reported in Table 1, most of the patients were male (n = 82; 76.6%), with a higher prevalence of treatment applied to the left hemisphere (n = 68; 63.6%). The disease duration was 9.23 ± 6.62 years, and the skull density ratio fell within the expected range for safe sonication (0.445 ± 0.086). LEDD data were available for a subset of patients at baseline and/or 6-month follow-up. Participant retention remained high throughout the study, with minimal attrition at each timepoint. Only a small proportion of participants were lost to follow-up, resulting in a balanced dataset across the four timepoints for the motor outcome measures (FTM and MDS-UPDRS-III). Cognitive measures showed a more variable pattern of availability, particularly for the MoCA, reflecting both planned omissions and occasional missing data.

**Table 1.** Baseline demographic and clinical characteristics of the study sample

Variable	Baseline	24 hours	1 month	6 months
Age (mean year ± SD)	69.365 ± 8.692	/	/	/
Sex (n)				
Male	82	/	/	/
Female	25	/	/	/
Disease duration (mean year ± SD)	9.237 ± 6.620	/	/	/
LEDD (n)	102	/	/	101
LEDD (mean mg/day ± SD)	479.892 ± 353.553	/	/	351.753 ± 256.112
Treated hemisphere (n)				
Right	39	/	/	/
Left	68	/	/	/
Skull density ratio	0.445 ± 0.086	/	/	/
Total number of sonications (n)	10.944 ± 2.823	/	/	/
Maximum energy (mean J ± SD)	14006.671 ± 7138.273	/	/	/
Maximum power (mean W ± SD)	737.292 ± 137.861	/	/	/
Maximum temperature (mean °C ± SD)	61.042 ± 3.338	/	/	/
Lesion volume (mean cm^3^ ± SD)	0.220 ± 0.097	/	/	/
FTM (n)	107	98	94	92
MDS-UPDRS-III (n)	104	96	95	92
MMSE (n)	59	59	47	42
MoCA (n)	94	/	78	67

Continuous variables are presented as mean ± standard deviation (SD); categorical variables are reported as absolute frequencies. Longitudinal data availability is reported for each outcome measure across the four timepoints (baseline, 24 hours, 1 month, and 6 months)

*FTM* Fahn-Tolosa-Marin Tremor Rating Scale; *LEDD* Levodopa Equivalent Daily Dose; *MDS*-*UPDRS*-III Movement Disorder Society Unified Parkinson’s Disease Rating Scale Part III; *MMSE* Mini-Mental State Examination; *MoCA* Montreal Cognitive Assessment; *SDR* Skull Density Ratio

### Motor outcomes

**Fig. 2.**
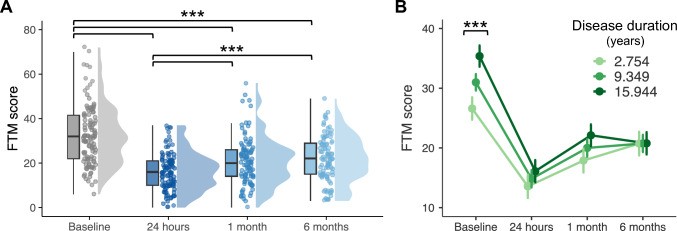
**A** Individual distributions, boxplots, and density plots of FTM scores before (baseline) and after MRgFUS treatment (24 hours, 1 month, 6 months). Asterisks show results of Holm-corrected post hoc comparisons (***p < 0.001). **B** Estimated marginal means (± standard error) of FTM scores across timepoints, plotted separately for three levels of disease duration derived from the sample mean ± 1 SD. Asterisks indicate significant prediction effect of disease duration on FTM score for individual timepoints (***p < 0.001). The statistical significance of the temporal pattern of treatment response is not reported in panel B as it was consistent across all levels of disease duration and resembled that shown in panel A. Abbreviations: FTM, Fahn-Tolosa-Marin Tremor Rating Scale

The analysis of FTM scores revealed a robust main effect of *Time* (F = 59.716, p < 0.001), indicating a significant change across the study period (Fig. 2A). Among the baseline covariates, none showed a significant main effect on FTM (all p ≥ 0.064).

Post hoc comparisons clarified the nature of the significant main effect of *Time*. FTM scores dropped sharply from baseline to the 24-hour post-treatment assessment (t = 12.995, p < 0.001), reflecting a substantial immediate reduction in tremor severity following MRgFUS. However, between 24 hours and 1 or 6 months, there was a significant increase in FTM scores (t = −3.932, p < 0.001; t = −4.591, p < 0.001, respectively), suggesting a partial rebound or early fluctuation in clinical response. Despite this increase, scores at both 1 and 6 months remained significantly lower than baseline (t = 8.511, p < 0.001; t = 8.122, p < 0.001, respectively), indicating a sustained therapeutic effect over time. Importantly, no significant change was observed between the 1-month and 6-month assessments (t = −0.565, p = 0.573), suggesting clinical stabilization during the later follow-up period.

Notably, a significant interaction was found between *Time* and *Disease duration* (F = 5.921, p < 0.001), indicating that the trajectory of FTM scores over time varied depending on the duration of Parkinson’s disease at baseline. No other interactions, including those involving *sex*, *age*, *treated hemisphere*, or *SDR*, reached significance (all p ≥ 0.655), suggesting a broadly consistent effect of the treatment across these subgroups. Further examination of the *Time* × *Disease duration* interaction effect (Fig. 2B) confirmed that the treatment led to a significant decrease in FTM scores over time across all subgroups (all p < 0.001), indicating a consistent beneficial effect of MRgFUS. However, the magnitude of this improvement varied depending on the duration of Parkinson’s disease. Participants with longer disease duration (EMM + 1 SD = 15.944 years) showed the largest reductions in FTM scores at all post-treatment timepoints (24 hours: −19.274, 1 month: −13.264, 6 months: −14.604). Conversely, those with shorter disease duration (EMM – 1 SD = 2.754 years) exhibited smaller improvements and a more attenuated trajectory (24 hours: −13.050, 1 month: −8.717, 6 months: −5.881). To further clarify the nature of the observed interaction, simple slope analyses indicated that disease duration significantly influenced baseline FTM scores (B = 0.665, p < 0.001), with longer disease history associated with more severe tremor. However, this relationship disappeared following treatment, becoming non-significant at all subsequent follow-ups (24 hours: B = 0.193, p = 0.307; 1 month: B = 0.320, p = 0.085; 6 months: B = 0.004, p = 0.984). Using a ≥30% reduction from baseline as a clinically meaningful response threshold, FTM response was observed in 85/98 patients (86.7%) at 24 hours, 58/94 (61.7%) at 1 month, and 52/92 (56.5%) at 6 months. In the complete-case responder-maintenance analysis, 78/90 patients (86.7%) achieved FTM response at 24 hours; among these initial responders, 51/78 (65.4%) maintained response at 1 month, 49/78 (62.8%) at 6 months, and 42/78 (53.8%) at both follow-up assessments.

**Fig. 3.**
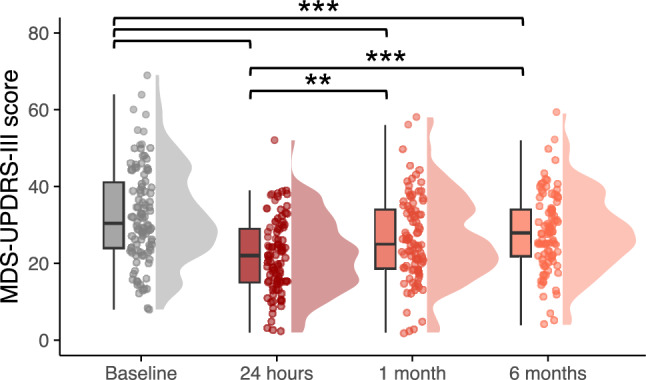
Individual distributions, boxplots, and density plots of MDS-UPDRS-III scores before (baseline) and after MRgFUS treatment (24 hours, 1 month, 6 months). Asterisks indicate results of Holm-corrected post hoc comparisons (**p < 0.01, ***p < 0.001). Abbreviations: MDS-UPDRS-III, Movement Disorder Society Unified Parkinson’s Disease Rating Scale Part III

As regards the MDS-UPDRS-III scale as dependent variable, a significant main effect of *Time* was observed (F = 34.861, p < 0.001), indicating that motor symptom severity changed significantly across the four timepoints (Fig. 3). Post hoc comparisons revealed a significant reduction in MDS-UPDRS-III scores from baseline to all subsequent timepoints (24 hours: t = 10.075, p < 0.001, 1 month: t = 6.196, p < 0.001, and 6 months: t = 4.952, p < 0.001). The largest reduction was observed between baseline and 24 hours post-treatment, with gradual attenuation of effects over time. Specifically, motor scores significantly worsened between 24 hours and 1 month (t = −3.515, p = 0.001) and between 24 hours and 6 months (t = −5.854, p < 0.001), indicating a partial rebound of motor symptoms. However, no significant difference was found between 1 month and 6 months (t = −1.235, p = 0.218), suggesting a stabilization of motor symptoms during the later phase of follow-up.

Among baseline covariates, *age* emerged as a significant predictor (F = 11.591, p = 0.001), suggesting that older individuals presented higher MDS-UPDRS-III scores across the study period. None of the other covariates, including *sex*, *disease duration*, *treated hemisphere*, or *SDR*, showed significant main or interaction effects (all p ≥ 0.227), indicating that the trajectory of MDS-UPDRS-III scores was broadly consistent across subgroups defined by these characteristics.

Defining clinically meaningful MDS-UPDRS-III response as a ≥30% reduction from baseline, response was observed in 58/96 patients (60.4%) at 24 hours, 37/95 (38.9%) at 1 month, and 26/91 (28.6%) at 6 months. In the complete-case responder-maintenance analysis, 53/90 patients (58.9%) achieved response at 24 hours; among these initial responders, 30/53 (56.6%) maintained response at 1 month, 22/53 (41.5%) at 6 months, and 22/53 (41.5%) at both follow-up assessments.

Additional analyses of MDS-UPDRS-III subdomains showed a significant main effect of Time for tremor (F = 51.248, p < 0.001), rigidity (F = 26.088, p < 0.001), bradykinesia (F = 17.046, p < 0.001), and axial symptoms (F = 2.841, p = 0.038). No significant Time × covariate interaction emerged for any subdomain (tremor: all p ≥ 0.202; rigidity: all p ≥ 0.089; bradykinesia: all p ≥ 0.162; axial symptoms: all p ≥ 0.235), indicating broadly consistent subdomain trajectories across baseline clinical and procedural characteristics. Age showed a significant main effect on tremor, bradykinesia, and axial scores (all p ≤ 0.037), but did not interact with Time.

Post hoc comparisons indicated that tremor scores decreased significantly from baseline to 24 hours, 1 month, and 6 months (all p < 0.001), with partial attenuation between 24 hours and later follow-up assessments, and no significant difference between 1 and 6 months. Rigidity scores decreased significantly from baseline to 24 hours (p < 0.001) but no longer differed from baseline at 1 or 6 months; scores were significantly higher at 1 and 6 months than at 24 hours (both p < 0.001), suggesting attenuation of the early effect. Bradykinesia scores decreased significantly from baseline to 24 hours (p < 0.001) and 1 month (p = 0.002), but not at 6 months; scores increased from 24 hours to 6 months (p = 0.004) and from 1 to 6 months (p = 0.032). For axial symptoms, although the omnibus effect of Time was significant, no pairwise comparison survived Holm correction, suggesting only a weak overall temporal modulation.

Finally, to explore whether motor improvement was accompanied by changes in dopaminergic treatment, we compared LEDD between baseline and 6-month follow-up in patients with available medication data. LEDD significantly decreased from baseline to 6 months (F = 19.992, p < 0.001), suggesting that clinical improvement was accompanied by a reduced need for dopaminergic treatment in this subset of patients.

Exploratory analyses including lesion-related and procedural variables (total number of sonications, maximum energy, maximum power, maximum temperature, and lesion volume) showed no significant Time × procedural parameter interactions for FTM (all p ≥ 0.430) or MDS-UPDRS-III trajectories (all p ≥ 0.190). Thus, within the available procedural measures, outcome trajectories were not significantly moderated by total number of sonications, maximum energy, maximum power, maximum temperature, or lesion volume.

### Cognitive outcomes

**Fig. 4.**
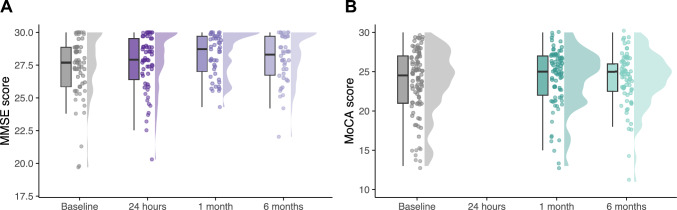
Distribution of MMSE (**A**) and MoCA (**B**) scores across the available timepoints MoCA was not included in the 24-hour follow-up. Abbreviations: MMSE, Mini-Mental State Examination; MoCA, Montreal Cognitive Assessment

As for the MMSE scores, no significant main effect of *Time* emerged (F = 1.272, p = 0.287), indicating that cognition remained stable from baseline to six months post-treatment (Fig. 4A). None of the baseline covariates (*age*, *sex*, *disease duration*, *treated hemisphere*, or *SDR*) showed significant main (all p ≥ 0.090) or interaction effects (all p ≥ 0.110), suggesting consistent trajectories among sample subgroups analysed.

Similarly, no significant effect of *Time* was observed on MoCA scores (F = 1.073, p = 0.345), suggesting that cognitive functioning remained stable across the follow-up period (Fig. 4B). None of the covariates significantly predicted MoCA scores (all p ≥ 0.079), except for *age* (F = 12.810, p < 0.001), with older participants showing lower scores irrespective of timepoint. Finally, analysis indicated that no significant interactions between *Time* and other covariates reached significance (all p ≥ 0.259).

### Adverse events

MRI/ultrasound-related adverse events included dizziness (5%), scalp burning (4%), headache (3%), and vasovagal reactions (1%). These events occurred during or immediately after the procedure and resolved spontaneously or with supportive treatment. Specifically, headache and scalp burning were managed conservatively without interruption of the procedure, while vasovagal reaction resolved after brief interruption of sonication and supportive measures. Dizziness was managed with supportive measures, including temporary interruption of sonication when required, hydration, and brief clinical observation. Symptoms resolved spontaneously, allowing the procedure to be completed in all patients.

Thalamotomy-related adverse events included paresthesias involving the face or upper limb (5%), contralateral weakness (4%), gait instability (3%), and dysgeusia (1%). These neurological symptoms progressively improved during follow-up, with gradual resolution within six months after MRgFUS thalamotomy. No serious adverse events, permanent neurological deficits, or procedure-related hospital readmissions were observed.

## Discussion

The present study evaluated the clinical outcomes of MRgFUS thalamotomy in patients with tremor-dominant PD. Importantly, the target population consisted of patients with highly disabling, medically refractory tremor, which represented the main criterion for treatment eligibility, irrespective of tremor severity scores, as no predefined threshold on tremor rating scales was required for access to MRgFUS. This approach is consistent with that suggested by current guidelines [2] and adopted in the most recent clinical trials, in which treatment eligibility was primarily based on the presence of disabling, medication-refractory tremor rather than on predefined tremor severity cut-offs [6]. Our findings showed a pronounced initial improvement in tremor after MRgFUS, followed by a mild deterioration at 1 month that subsequently stabilized at 6 months. This pattern suggests that most of the benefit occurs immediately after the intervention, with partial attenuation of the effect across six months.

The subdomain analysis of the MDS-UPDRS-III clarified that the improvement in the total motor score was mainly driven by tremor-related items, as expected given the VIM target of MRgFUS thalamotomy. This pattern is consistent with the target-specific effects of MRgFUS, as VIM thalamotomy primarily modulates the cerebello-thalamo-cortical tremor network, whereas MRgFUS targeting the subthalamic nucleus has been shown to produce broader improvements in parkinsonian motor features, including bradykinesia and rigidity [23]. In our patients, short-lived improvements were observed for rigidity and bradykinesia, although these effects attenuated over follow-up, whereas axial symptoms showed only a weak temporal modulation. This pattern supports the interpretation that MRgFUS primarily acts on tremor, while changes in non-tremor motor domains should be interpreted cautiously and may reflect indirect effects, clinical variability in medication-OFF assessments, or reduced interference of severe tremor with motor examination rather than a broad disease-modifying motor effect.

In our cohort, longer disease duration was associated with greater baseline tremor severity. This finding should be interpreted with caution, as it may, at least in part, reflect selection bias. Indeed, patients referred for MRgFUS are often older, have a longer disease duration, and present with persistent, disabling tremor despite optimized medical therapy. Therefore, this association is likely to reflect the characteristics of the selected study population rather than the natural longitudinal evolution of tremor severity in PD, which is heterogeneous and may fluctuate, decrease, or evolve over time. However, patients with longer disease duration, in our cohort, exhibited larger early improvements at 24 hours following MRgFUS, resulting in comparable tremor severity across disease-duration histories at all follow-up timepoints. The fact that disease duration was associated with tremor severity at baseline but not at post-treatment timepoints suggests that MRgFUS may mitigate the impact of disease chronicity on tremor severity, resulting in a more uniform post-treatment clinical profile among patients with varying disease durations. The safety profile of MRgFUS was favorable, with no serious adverse events, permanent neurological deficits, or procedure-related hospital readmissions. All procedure- and thalamotomy-related adverse events were transient, managed conservatively when required, and resolved within the 6-month follow-up period. The incidence of adverse events in our cohort was lower than that reported in some previous studies [6, 7]. Although direct comparisons should be interpreted with caution because of differences in study design, patient populations, and adverse event reporting, the progressive refinement of the MRgFUS technique, including the learning curve, improvements in patient selection, and optimization of treatment planning, may have contributed to the favorable safety profile observed in our cohort. Indeed, previous work from our group demonstrated that increasing operator experience was associated with improvements in procedural performance and clinical outcomes, suggesting that progressive refinement of the technique may also contribute to reducing treatment-related adverse events [24].

Notably, the robust efficacy observed in the overall sample, together with the post-treatment convergence of tremor severity across disease-duration histories, supports MRgFUS as a potential therapeutic option at all stages of the disease. Unlike other advanced therapies, which are primarily indicated for the management of motor fluctuations and dyskinesias, MRgFUS and DBS share a specific indication for medically refractory tremor. However, as compared to MRgFUS, DBS is often preferentially considered in younger patients with a shorter disease duration [2, 25, 26]. Moreover, MRgFUS may be temporally integrated with DBS, which generally follows rather than precedes MRgFUS, unless DBS electrodes have been removed or MRI-compatible hardware has been used [27, 28]. This helps to overcome the controversy surrounding DBS and MRgFUS as direct competitors, suggesting instead that they should be viewed as complementary procedures, as together they may offer a more comprehensive therapeutic approach than either modality alone [29]. In particular, combining the static effects of MRgFUS with the dynamic, adjustable effects of DBS may offer a synergistic strategy for more individualized management of tremor. Our findings also suggest that MRgFUS may facilitate a reduction in LEDD, potentially postponing the need for other advanced therapeutic interventions. Our patients may have undergone escalation of dopaminergic therapy before MRgFUS because of disabling, medically refractory tremor. The subsequent reduction in tremor may have allowed de-escalation of pharmacological therapy, as reflected by the significant decrease in LEDD observed during follow-up in our cohort, consistent with findings of other studies [30, 31].

The strengths of our study include its longitudinal design, which allowed the collection of follow-up data over time. In addition, the availability of cognitive assessments at multiple time points in a substantial proportion of patients enabled evaluation of the cognitive safety profile of MRgFUS. While recent multicentre studies and meta-analyses have reported findings from similarly large cohorts, often with longer follow-up [6–8], our results provide independent confirmatory evidence from a single-centre cohort and further support the reproducibility of the clinical and cognitive outcomes associated with MRgFUS in tremor-dominant PD. The main limitation of our study is the absence of a control group, which precludes a definitive distinction between the treatment effect and potential placebo responses. Although randomized sham-controlled trials represent the gold standard for evaluating treatment efficacy and have been successfully conducted in patients undergoing MRgFUS thalamotomy [6], the currently available randomized evidence remains limited to relatively small cohorts, and the implementation of such studies continues to be associated with important ethical and practical challenges. Participants allocated to the sham arm are required to undergo hospitalization, complete scalp shaving, and prolonged immobilization within the MRI scanner despite receiving no therapeutic intervention. Nevertheless, several observations suggest that the clinical improvement observed in our cohort is unlikely to be explained entirely by placebo effects, regression to the mean, or the natural fluctuation of tremor severity. Although placebo responses in tremor-dominant PD have been demonstrated experimentally, they are heterogeneous and predominantly short-lived [6, 32]. Therefore, placebo effects alone are unlikely to account for the sustained improvement observed after MRgFUS, particularly when considered together with the significant reduction in LEDD during follow-up. Indeed, in our cohort, the significant reduction in LEDD during follow-up suggests that pharmacological therapy was not intensified to achieve tremor control and may instead have been de-escalated following the clinical benefit associated with MRgFUS. Other limitations of this study include the relatively short follow-up, which precludes conclusions regarding the long-term persistence of the observed clinical benefits and may not have captured late tremor recurrence. In addition, the assessment of overall tremor severity, rather than separate analyses of resting and postural/action tremor, precludes conclusions regarding the differential effects of MRgFUS across specific tremor subtypes. Furthermore, cognitive outcomes should be interpreted with caution, as assessments were based on screening instruments rather than comprehensive neuropsychological batteries, and complete longitudinal cognitive data were not available for all patients. More detailed assessments, particularly of executive functions and other specific cognitive domains, would have provided a more sensitive evaluation of the cognitive effects of MRgFUS. Nevertheless, they are consistent with our previously published prospective studies [11, 12] based on comprehensive neuropsychological assessments and longer follow-up, and thus provide further confirmatory evidence supporting the cognitive safety profile of MRgFUS in patients with tremor-dominant PD. Additional limitations that may affect the generalizability of the findings include the monocentric design of the study and the relatively low number of female participants. This imbalance may reflect the lower prevalence of the disorder in women, as well as potential sex-related differences in referral patterns to advanced therapies [33]. Finally, although we explored available procedural parameters and lesion volume, we did not perform detailed lesion-location analyses based on stereotactic coordinates, tractography, or voxel-wise lesion mapping. This represents an important limitation, as recent evidence suggests that lesion topography and targeting accuracy may be more informative than global procedural parameters or lesion size in explaining tremor relapse and long-term response variability [9, 10]. Further longitudinal studies with extended follow-up and detailed lesion-location analyses are needed to clarify the long-term effects of MRgFUS on tremor and the factors underlying response variability and potential relapse. Finally, future studies should explore the potential of staged repeated thalamotomy and bilateral staged MRgFUS to consolidate therapeutic benefits, given the currently limited evidence regarding the safety and efficacy of bilateral lesioning in PD.

## Data Availability

The datasets generated and analysed during the current study are available from the corresponding author on reasonable request
